# Drinking guidelines: balancing between science and popular opinion

**DOI:** 10.1093/eurpub/ckae194

**Published:** 2025-04-03

**Authors:** Sven Andreasson

**Affiliations:** Karolinska Institutet, Stockholm Centre for Dependence Disorders, Stockholm, Sweden

In their Viewpoint article, Kevin Shield and Saverio Stranges [1] discuss official recommendations regarding alcohol consumption. Their view is that these go too far when they advocate for universal total alcohol abstinence. This seems reasonable but the question is where such advice is given. Their example is the Nordic Nutrition Recommendations 2023, published by the Nordic Council of Ministers [2]. These recommendations certainly are quite restrictive, but they do not advocate for total abstinence. What they do say is the following: “Based on the overall evidence, it is recommended to avoid alcohol intake. If alcohol is consumed, the intake should be very low.” This message is consistent with recent epidemiological research, where no safe limits for drinking are found. While the jury is still out regarding possible positive effects from very low consumption on cardiovascular disease, these effects are outweighed by the negative effects on other diseases and trauma. For certain vulnerable groups, the recommendation is more categorical: “For children, adolescents and pregnant women abstinence from alcohol is recommended.” There appear to be no official guidelines that recommend universal abstention. 

There are two types of guidelines. One is guidelines for professionals in health care, intended to support practitioners in choosing when to offer advice and the content of this advice. In the real world of medical practice, it is necessary however to take into account a number of circumstances, medical and social, which may call for a modification of the advice given.

The other is guidelines for the general public. These are by necessity general in nature and are motivated by the public’s right to information. They are often requested by interest groups, the media, and decision-makers in health and social affairs. Here it is important to underline the general nature of the guidelines and that there are important exceptions when abstinence should be advised. While pregnancy and driving are well recognized indications for abstinence, few guidelines mention mental illness, carrying a weapon or similar situations not compatible with alcohol use.

Shield and Stranges seem to have misinterpreted the Nordic Nutrition Recommendations. But even if there appear to be no examples of official guidelines advocating universal total alcohol abstinence, the question remains whether restrictive guidelines can backfire by causing public distrust in researchers and authorities.

Perhaps this concern is exaggerated. There appears to be a growing understanding in the general public, at least in western countries, that drinking alcohol is unhealthy, even when consumption is moderate. One may choose to drink anyway, but less. This appears to be the trend in many countries, exemplified by a recent Gallup poll in the USA [3], where one finds that an increasing proportion of young adults now find even moderate consumption unhealthy. Fifty-two percent held this view in the survey this year, an increase by 18 percentage points compared with the same survey in 2019. Among middle aged, the study found a smaller increase, at 13%, and among the elderly no change.

The larger question is what role the authors of drinking guidelines have. To report as accurately as possible what the risks from alcohol are—or compromise with prevailing attitudes. It should be clear that the drinking guidelines should report what the actual health risks are, with the logical conclusion that from a health point of view consumption should be minimized. This is the message from the Nordic Nutrition Recommendations 2023, and the 2023 Canadian Guidance on Alcohol and Health. This does not preclude that alcohol drinking can convey other benefits, social and cultural, that may outweigh the medical risks for the individual consumer. Consideration of other benefits should not contaminate the medical assessments however. People may drink, but should not be deluded to think that drinking is risk free. This line of thinking is what prevails in advice for other health habits. You may enjoy your beef burger, without having been told by some guideline that it is ok from a health point of view to do so.

Conflict of interest: None declared. 
